# Curriculum-based sexual and reproductive health education: revealing its relevance for risky sexual behaviors among secondary school students in Mwanza, Tanzania

**DOI:** 10.1186/s12978-024-01798-x

**Published:** 2024-04-29

**Authors:** Ng’wamba Sitta Ngissa, Erica Sanga, Mussa Kelvin Nsanya, Belinda Kweka, Evangelista Malindisa, Rosemarie Mwaipopo

**Affiliations:** 1 Department of Clinical Research Program, National Institute for Medical Research, Mwanza, Tanzania; 2 Department of Physiology, Catholic University of Health and Allied Sciences, Mwanza, Tanzania; 3 Department of Anthropology, University of Dar es Salaam, Dar es Salaam, Tanzania

**Keywords:** Curriculum-based sexual and reproductive health, Risky sexual behaviors, Secondary school students, Relevance, Tanzania

## Abstract

**Background:**

Secondary school students are vulnerable to risky sexual behaviors (RSBs) which may lead to adverse health consequences, such as teenage pregnancies and sexually transmitted diseases (STDs), including HIV/AIDS. In Tanzania, the burden of teenage pregnancy was reported to be 27% in 2016. The integration of sexual and reproductive health (SRH) education into the school curriculum is one of the proven crucial interventions. However, there is limited information on the extent to which curriculum-based SRH education is relevant for fostering good practices for coping with RSBs. This study sought to describe students’ and teachers’ perceptions of the relevance of curriculum-based SRH education.

**Methods:**

A qualitative study was conducted from May to June 2020 (involving 5 secondary schools in Ilemela district, Mwanza, Tanzania). In-depth interviews (30) were conducted among secondary school students and 10 interviews for teachers. The data were collected in Swahili and then transcribed and translated into English after which thematic content analysis was performed.

**Results:**

The majority (56%) of secondary school students were revealed to have a limited understanding of curriculum-based SRH education, which was limited to a few aspects of health that involved married people and pregnant women. Teachers of different subjects had different perceptions about the relevance of curriculum-based SRH education. Civics teachers had the perception that it was relevant and enough, while Biology teachers thought that it was not enough. Students reported utilizing the information taught in class to manage and navigate RSBs. Moreover, they expressed a need for additional delivery strategies to be used for a comprehensive understanding of sexual and reproductive health.

**Conclusion:**

Despite the identified gaps in providing a comprehensive knowledge that builds on the appropriate attitudes and skills, the existing curriculum-based SRH education in secondary schools was utilized to help students in addressing and managing RSBs. However, there is a need for more comprehensive information and an improved delivery approach for SRH to equip students with the necessary skills when faced with RSBs.

## Abstract (plain language summary)

Sexual and reproductive health (SRH) education integrated into school curricula has proven effective in addressing risky sexual behaviors (RSBs) among in-school adolescents. Adolescents attending secondary schools, are susceptible to RSBs such as early sexual activity, multiple partners, unprotected intercourse, and unsafe abortions, leading to adverse health outcomes such as teenage pregnancies and HIV/AIDS. Despite the integration of SRH education into secondary school syllabi, these behaviors are still evident among students. There is a lack of data on the impact and relevance of curriculum-based SRH education on RSBs. To explore the relevance and effectiveness of SRH education in addressing RSB patterns, in-depth interviews were conducted among secondary school students and civics and biology teachers focusing on their perceptions and practical outcomes of SRH education in reducing RSBs. After conducting a thematic content analysis three main themes were discovered: (1) Student’s understanding of curriculum-based SRH education in coping with RSBs (2), Perceptions of curriculum-based SRH education in coping with RSBs and (3) Utilizing curriculum-based SRH education in coping with RSBs. Majority of secondary school students (56%) had a limited understanding of SRH narrowed to aspects concerning married individuals and pregnancy. Teachers provided varying opinions regarding the efficacy of curriculum-based SRH education, with Civics instructors believing there is relevance while Biology teachers considered it limited. Students acknowledged using the available knowledge to address RSBs but expressed a desire for supplementary strategies to achieve a more thorough understanding of SRH. Improving the SRH education provided in schools will help reduce the consequences of RSBs.

## Introduction

Sexual and reproductive health (SRH) education integrated into the school curriculum is an effective intervention for in-school adolescents before risky sexual behaviors (RSBs) are firmly established [1–3]. Adolescents aged 10–19 years are estimated to constitute 16% and 23% of the global population and the Tanzania population, respectively, with 90% of them living in low-middle-income countries (LMICs) [4–7]. Adolescents are vulnerable to RSBs, including having sex at an early age, having multiple sexual partners, having sex while under the influence of alcohol or drugs, having unprotected sex, and having unsafe abortions [8, 9]. Such behaviors may lead to adverse health consequences, such as teenage pregnancies and sexually transmitted diseases (STDs), including HIV/AIDS. For instance, in 2010 and 2016, an increase in teenage childbearing from 23% in 2010 to 27% in 2016 was reported in Tanzania [10].

Curriculum-based SRH education encompasses the contents taught in the classroom following the guidelines set forth by the secondary school syllabus prepared by the Tanzania Institute of Education (TIE). It covers various topics such as anatomy, physiology, reproductive processes, contraception, sexually transmitted infections (STIs) and healthy relationships. The curriculum-based approach ensures that SRH education is systematically integrated into academic programs, typically in secondary schools or higher education institutions. The importance of the provision of SRH education in the school setting has affected youths from a global, national and individual level in reducing the risks associated with the practice of RSBs. Schools may often be the only place where adolescents can obtain accurate information on reproductive health [11].

In 2005, TIE issued a secondary school syllabus in which sexuality content was integrated and offered along with other subjects, such as Home Economics, Biology, General Studies, and Civics, in secondary schools [12]. The syllabus content mainly focused on human reproductive anatomy, conception, sexually infectious diseases, risk behaviors, and communication skills [13]. For instance, Biology mainly focuses on general health and well-being when it comes to SRH while Civics covers the women’s role in society, economy, laws and policies relating to reproduction and reproductive health services [13–15]. Evidence shows that integrating comprehensive SRH education into secondary school curricula could play a vital role in enhancing adolescents’ understanding of sexuality and addressing their reproductive health concerns [16].

Despite the efforts invested in by TIE, secondary school students still practice RSBs [17]. For instance, in recent years, Mwanza city has consecutively ranked first in Tanzania, with the relatively highest proportion of secondary school dropouts due to pregnancy being 10.8% and 9% in 2018 and 2019, respectively [18]. Information regarding the impact of curriculum-based SRH education in secondary schools is not well documented, and data regarding perceptions of curriculum-based SRH education provided to secondary school attending adolescents are scarce [1]. In addition, local data on the extent to which curriculum-based SRH education has translated to reducing RSBs are scarce.

The aim of this study was therefore to examine the relevance of curriculum-based SRH education for RSB patterns among secondary school students in Mwanza, Tanzania. Specifically, we aimed to assess secondary school students’ and teachers’ perceptions of curriculum-based SRH education and how curriculum-based SRH education has helped students cope with RSBs.

## Methods

### Research design and study area

A qualitative cross-sectional study was conducted from May to June 2020 in the Ilemela District of the Mwanza region in northwestern Tanzania. Ilemela district is located between latitude 2.4485^0^ South and 32.9663^0^ East in Mwanza region in north-western Tanzania. The district is administratively divided into 19 wards with a human population of 480,779 and a population density of 1,347 per km^2^ with an annual growth rate of 2.6%, and there are 50 secondary schools. According to Basic Statistics Tanzania (2019), the number of secondary school students in Ilemela District secondary schools was estimated to be 31,124. A qualitative approach was appropriate for capturing students’ and teachers’ perceptions of curriculum-based SRH education and examining how such education has helped students cope with RSBs. To clarify the relevance of the existing patterns of RSBs, this study involved selected secondary schools in Ilemela District.

#### Selection of schools

We purposively selected 5 secondary schools, which included public/privately owned, day/boarding, and single-sex/coeducation schools. This approach aimed to understand the perceptions of curriculum-based SRH education and coping strategies of RSBs among students with diverse backgrounds.

#### Selection of classes

The target population for this study was Form III and IV secondary school students who provided information on how curriculum-based SRH helps them make proper decisions when dealing with RSBs. Secondary school students at these class levels already have more than 50% of their required study years (4 years) and hence have great coverage in subjects.

#### Selection of students

In each school, 6 (3 males and 3 females or 6 students in single-sex schools) secondary school students were invited for interviews. Interviewed secondary school students were obtained by randomly selecting males and females from Form III and Form IV classes. A total of 30 students from 5 secondary schools in the Ilemela District (Mwanza) were invited to participate in this study.

#### Selection of teachers

We purposively selected and interviewed a total of 10 civics and biology teachers (2 from each school). We chose these two subjects because they had content for curriculum-based SRH education. Hence, teachers provided information on what the curriculum comprised about the coverage of SRH education curriculum content and how it was supposed to be delivered.

### Data collection procedures

We separately conducted a total of 40 in-depth interviews among students (30 interviews) and 10 among teachers using interview guides, checklists, and tape recorders. The interview guides were prepared in the English language and subsequently translated to the Swahili language, which is the native language for most of the participants. The average time taken for interviews with students and teachers was 30 min. To ensure that the translated interview guide had the same meaning in both languages, it was translated back into English and still had the same meanings. Two research assistants proficient in conducting interviews, interviewed the study participants in Swahili language which is a native language for most of the participants. To ensure validity, the interview guides were pre-tested before the data collection procedures. Interviewers reviewed what the participants said to ensure clarification of anything unclear or ambiguous.

### Data analysis procedures

The analysis and interpretation of findings were based on three broad themes. Under each theme, several subthemes emerged, as shown in Table 1 below.

**Table 1:** Summary of themes and subthemes

	Themes	Sub-themes
1.	Student’s Understanding of Curriculum-Based SRH Education in Coping with Risky Sexual Behaviors.	Understanding of Sexual and Reproductive Health related contents.
		Understanding of Risky Sexual Behaviors related contents.
2.	Perceptions on Curriculum-Based SRH Education in Coping with Risky Sexual Behaviors.	Teachers’ perception of relevance of curriculum-based SRH education on RSBs.
		Teachers’ perceptions of the suitability of subjects for SRH contents addressing RSBs.
3.	Utilizing curriculum-based SRH education in coping with RSBs.	Students’ SRH education application from curriculum-based SRH education
		Influence of teaching methods in establishing relevance

### Ethical consideration

Ethical clearance was issued by The University of Dar es Salaam with reference number AB3/12(B). Additionally, the Ilemela District municipality provided a permit to conduct this study in the selected secondary schools. Provided that curriculum-based SRH education is a sensitive issue in the Tanzanian context, throughout this study, the investigators were careful in verbal and nonverbal language to avoid any physical or emotional harm. Thus, participants were well informed in advance of the research problem under investigation and were told that they were free to participate or withdraw from the study at any time. Legal guardians/parents of participants below the age of 18 were given written informed consent. Assent was obtained from participants below the age of 18 before interviews. Prior to conducting the interviews, participants were briefed on the benefits of this research for the education sector and youth well-being. Participants were also reassured that participating in the interviews posed no risks. Moreover, participants received additional information regarding questions related to SRH and RSBs after the interviews were completed. Verbal and written consent from all study participants was sought and participants consented to the publishing of their responses anonymously. Only participants who consented to participate in the study were included. Confidentiality was also observed, as were cultural values, traditions, and taboos.

## Results

### Students’ understanding of curriculum-based SRH education in coping with risky sexual behaviors

#### Understanding of curriculum-based SRH education content

Many of the respondents (female and male students) gave vague ideas on what they knew about SRH. In the interviews, SRH was described from various perspectives based on the type of school the respondent attended. One of the respondents stated:

*“Sexual and Reproductive Health is a state where both men and women have awareness and well-being on their health in regards to their sexual and reproductive health. For example, people may be able to treat and protect themselves from diseases affecting the reproductive system.”* (Male, Form IV student, private boarding coeducation school)

However, many of the female students were shy about providing their understanding of SRH which could be attributed to culture and norms, as some respondents admitted that they had no idea what SRH was. As one of them said.

*“..I am truly not sure what SRH is…”* (Female, Form III student, private day coeducation school)

Another subtheme on the meaning and awareness of SRH that was mentioned was the issue of pregnancy and family planning, which are associated with the issues that are involved in SRH education. This was a common response in single-sex schools, as one of the male respondents indicated that SRH is a matter that involves women.

*“It is the health during and after pregnancy that helps women to have a safe delivery.”* (Male, Form IV student, public boarding single-sex school)

#### Understanding of risky sexual behaviors contents

The majority of respondents, especially those from single-sex boarding schools, reported that RSBs are behaviors that, when practiced, may negatively impact the SRH of an individual. For students who had information on what RSBs are in particular, they would identify them in general what they are, while others could give examples of what they regard as RSBs. For instance, the respondent would say,

*“…behaviors such as having sex while at school, not using a condom and having sexual relationships with many girls for boys and many boys for girls are RSBs to a student”* (Female, Form IV students, private boarding single-sex school).

*“…they are dangerous behaviors that lead to health disorders, particularly those of the reproductive system.”* (Male, Form III student, public boarding single-sex school).

However, a few participants agreed that they knew what RSBs are but gave meaning that indicated unawareness of the related meaning of RSBs. A respondent in one of the schools said:

*“These are behaviors that are done without one’s awareness.”* (Male, Form IV student, Public day coeducation school)

#### Teachers’ perceptions of the relevance of curriculum-based SRH education for RSBs

Civics and Biology teachers also gave their opinions on whether curriculum-based SRH is relevant to students’ SRH knowledge and whether it enables them to cope with RSBs. Most of the Civics teachers explained that content coverage is sufficient to equip students with better decision-making about RSBs. The respondent (civics teacher) insisted that:

*“…based on their levels, the information is quite enough for this level, and as they keep advancing to other levels more will be revealed depending on the type of setting, they will be in, but in this context, the information is enough and helpful.”* (Female, Civics Teacher, private-boarding single-sex school)

The majority of biology teachers expressed that the information was too general to have any effect on RSB practices. One of the respondents (a biology teacher) replied,

*“It is a sensitive topic, but the education is provided to accomplish the syllabus requirements. With such general information, it is not possible to affect a large number of students.”* (Female, Biology Teacher, private-boarding single-sex school)

#### Teachers’ perceptions of the suitability of subjects with SRH contents for addressing RSBs

In interview sessions with civics and biology teachers, various opinions were given about whether the issues covered were suitable for providing students with knowledge of their SRH and changing their patterns of RSB practices. The relevance of these issues seems to vary among teachers in public and private schools.

*“… all that is covered in Civics makes a great contribution to students since the provided guidance on teaching such issues makes it easier for teachers to deliver the information with relevant pieces of evidence from the media and everyday events.”* (Male, Civics Teacher, Public boarding single-sex school)

On the other hand, most teachers in private schools believe that more needs to be done for the messages to be easy to understand; additionally, they suggest the following:

*“Speaking of their relevance, I think it is quite complicated, as the contents seem to be overwhelming to students. Biology is quite broad, so I cannot guarantee that the issues covered are relevant enough to make an impact on their life in general. The general concepts including the human reproductive system, genetics, and the menstrual cycle… I would not say that are relevant as the content is not detailed enough for them to utilize the entire information related to RSBs.”* (Male, Civics Teacher, private boarding single-sex school)

### Utilizing curriculum-based SRH education in coping with RSBs

#### Students’ application of curriculum-based SRH education

Practical application of curriculum-based SRH education was revealed to contribute to some aspects of pregnancy and STDs only. Students expressed their fears for the outcomes associated with practicing RSBs. The respondents specified that.

*“From what I understood in class, the effects of having bad behaviours (referring to RSBs ) may lead to pregnancy and diseases that may take a long time to cure. …. especially the issue of pregnancy at this age is what scares me the most.”* (Female, Form IV student, private day coeducation school)

Another respondent added the following:

*… we understand the outcomes of RSBs, and most of them may completely change one’s life. I am more interested when we are told of examples that make me think about my circumstances and I end up not doing it though sometimes it is hard to escape some mob influences.* (Male, Form IV student, public boarding single-sex school)

#### Influence of teaching methods in establishing relevance to RSB outcomes

##### Teachers

Teachers use various means to help deliver messages about SRH and show its relevance to RSB outcomes. One of the respondents (Biology Teacher) stated:

*“…being friendly to students helps facilitate open discussions on issues that are myths received from other sources such as their fellow peers. As I teach, students are so cooperative in regard to the reproduction topic; thus, the issues I address are well understood.* (Female, Biology Teacher, private boarding coeducation school)

On the other hand, the use of practical illustrations was mentioned by respondents as effective in delivering SRH education. The respondent (Civics Teacher) said,

*“…sometimes diagrams in textbooks are not enough to explain how the RSBs can have negative consequences on one’s life. I prefer using references from life stories and drawing lessons from movies such as that of “Yellow Card” to help them have a clear picture of the effects of RSBs.”* (Male, Civics Teacher, Private day coeducation school)

##### Students

When asked about how education on SRH is given to students, students are informed about how their teacher delivers such information. Some responded that their teachers use pictures/diagrams once in a while film was used and that discussions involved questions and answers. One of the respondents specified,

*“In Civics, our teacher uses live stories from the community emphasizing how we should apply life skills.* (Female, Form IV student, Public day coeducation school)

In another school where some respondents were also interviewed, they explored how they were taught about the effects of SRH and RSBs. The respondent stated,

*The teacher just talks by providing explanations following what is written in textbooks.* (Male, Form III student, Public day coeducation school)

Secondary school students believed that the information provided by trained facilitators from nongovernmental organizations (NGOs), such as FEMA, on SRH matters is different from that provided in class. In classroom lessons, curriculum-based SRH education is delivered following what the syllabi instructs, while the information given by trained facilitators from NGOs from various programmes visiting the school consists of extensive information. Among the respondents providing this information, one mentioned the following:

*“…in class, some teachers are open enough, though when experts visit, they are more open to giving further information, and most students get comfortable as they are friendly and have no exam requirements from such programs.”* (Female, Form III student, private boarding coeducation school)

## Discussion

This study examined the relevance of curriculum-based SRH education for coping with RSBs among secondary school students in Mwanza, Tanzania. The findings reveal gaps that reduce the relevance of curriculum-based SRH education. Culture and how SRH knowledge is delivered plays a significant role in shaping the approach to RSBs. The curriculum’s relevance primarily influences student and teacher perceptions of utilizing SRH education to address RSBs.

We have revealed that knowledge of curriculum-based SRH education is limited to a few aspects, such as family planning, pregnancy issues, and topics related to women or married individuals. The explanations given were observed among most male students, especially those in single-sex schools. Students in single-sex schools were revealed to have a limited understanding which is limited to matters that mostly involve women e.g., pregnancy, childbearing and family planning. This limited understanding of SRH leaves young people exposed and vulnerable to reproductive health consequences, thus emphasizing the need for comprehensive education that can influence behavioural changes [19]. The limited understanding can be attributed to unfriendliness among teachers and students since teachers can be perceived as critical to students’ issues. This would lead to resorting to wrong sources of information on sensitive issues that involve their SRH. More effort is to be made in combating RSBs among students by encouraging better communication between teachers and students. Additionally, the delivery of information about SRH varies depending on how a particular school can afford the infrastructure such as illustration samples for SRH topics e.g.; contraceptive materials and diagrams. The use of various means used in teaching SRH has varying impacts on students’ cognitive abilities. On the other hand, female students revealed to be uncomfortable and shying away from discussing SRH issues. This accounts for the existence of cultural and religious practices that make such discussions uncomfortable. For instance, the cultural practices that provide informal SRH education, such as “Jando” and “Unyago”, are informal SRH sources of information in some regions of Tanzania [20]. Curriculum-based SRH education needs to enrich students’ knowledge by adhering to standards influencing comprehensive and rights-based programs that convey clear messages that are appropriate for age, sexual experience, gender, and culture. An important starting point is their knowledge, misconceptions, hopes, and fears [21]. The provided explanations are limited to only meanings and a few aspects and the inability to speak about SRH matters implies that the achievement of needed skills to cope with RSBs is impossible because guidance on developing practical skills is absent. The curriculum-based SRH education has a gap in matters concerning emotions and self-regulation that are not taught and applied, without addressing these aspects, individuals may struggle to resist situations that could potentially lead to engaging in RSBs. Essential information is provided at later stages i.e. Form III and IV, making it challenging to effectively address RSB practices that may already be occurring. This leaves students with the role of fulfilling the obligation of studying the content for examination and pass mark purposes. Moreover, curriculum-based SRH education has been revealed to provide a glimpse of basic knowledge rather than intensive education that will impact students’ decision-making skills, attitudes, and knowledge. Given that this information becomes more intensive in later levels i.e. Form III and Form IV, the timing becomes quite too late as by this time puberty has already kicked into a majority of secondary school students.

The difference in perceptions of the relevance of curriculum-based SRH education among civics and biology teachers reveals a gap in the content taught. Biology teachers believed that the content being taught was restrictive and did not allow for practical application, which could hinder students from understanding the relevance of the subject matter. Civics teachers’ perceptions of what is being taught suggest that the moral aspects, mostly covered in civics, are seen as complementary to the SRH information taught in other subjects. The gap becomes evident in the syllabi, where although they include information on SRH, students receive minimal knowledge, primarily focused on attaining passing grades rather than understanding the content. Analysis of secondary school curricula content concerning SRH Education, particularly addressing RSBs, has uncovered limited and generalized information. This approach leaves out crucial skills necessary for youths to effectively address RSBs. Teachers’ perceptions about the relevance of information presented in the curriculum give rise to concerns that, given their daily interactions with students, it is important to acknowledge that there might be significant instructions on coping with RSBs and SRH information that are overlooked. A qualitative study done in Nigeria also found that the teaching of biology and integrated science in the curriculum may not be sufficient for the students since the courses may have limitations of impacting the desired life skills and may have other challenges such as the availability of adequate class time and appropriateness of the class setting for effective results [22]. Depending on the teachers’ perceptions, students are likely to have unequal information about SRH education, which may affect their ability to cope with RSBs. Moreover, determining whether content coverage is effective can be strongly affected by the teacher’s comfort, values, and competence in delivering such information given that it is considered sensitive in this context. Thus, teachers require training in SRH education delivery to ensure that consistent information is provided to every student.

Private schools have taken initiative to employ alternative tactics to emphasize the application of SRH education, aligning content with existing RSBs patterns [23]. Teachers, as reliable sources of information in schools, are expected to deliver SRH education by employing various techniques and demonstrating skills that are required for effective addressing of RSBs. Teachers’ interactions with students are among the essential components that create comfortable environments for both teachers and students. UNESCO emphasizes that “teachers responsible for the delivery of comprehensive sexuality education also require training on the specific skills needed to address sexuality accurately and clearly, as well as the use of active, participatory learning methods” [24]. However, culture also influences the ability of teachers to open up talking about SRH to students. This is in line with a review of country experience in Sub-Saharan Africa which found that teachers face pressure to focus on examinable subjects, often at the expense of life skills education. Evaluations of life skills education programs revealed teachers are often inadequately trained to deliver the lessons and may feel uncomfortable discussing sexuality issues. Thus, they may not teach them or schools may encounter resistance from religious groups when addressing sexuality education that deviates from a conservative, abstinence-only approach [25]. Teachers, therefore, need to facilitate students’ ability to combine and integrate what they learn in school with other knowledge disciplines if they are to develop effective decision-making skills [26]. The Education Sector Development Plan highlights its Logical Framework Outcomes, which include the Quality of Basic and Secondary Education. The expected result is that teacher training colleges have the necessary facilities and resources, employ innovative ways to help students understand science subjects and draw more secondary teachers to better prepare them [27]. With such a plan, the quality of SRH education can be assured, although employing other forms of teaching materials depends on the school’s ability to have the infrastructure that supports the use of such materials. This reflects that when there are no or limited resources, students become deprived of SRH knowledge to address RSBs.

Secondary school students show greater excitement towards events that promote health, such as bonanzas. The presence of external facilitators in schools facilitates the delivery of accurate and precise information. When participating in these activities, students are more likely to openly share their thoughts and feelings, as they feel confident that the school will not take disciplinary action against them, unlike when confiding in teachers. This reveals that subject teachers are limited to talk about SRH in classroom interactions since it is part of the curriculum requirement. This in turn perpetuates the practice of training students to pass examinations rather than getting them interested and engaged in the subject. The encouragement of such events supported by NGOs is useful because they also fit within the established guidelines on delivering SRH information. The provision of curriculum-based SRH in schools has revealed that students depend on such information to make decisions concerning the practice of RSBs.

### Study limitations

This study contributes to the emphasis placed on the significance of curriculum-based SRH education in reducing the health consequences faced by adolescents due to RSBs. Through the revelation of gaps in the contents, how the education itself is delivered and the relevance of SRH education in addressing RSBs; the study contributes to areas that call for improvement in the education sector which is the reliable source of information. Moreover, to the best of our knowledge, this study is among the few studies that provide the status of curriculum-based SRH education relevance to secondary school students in coping with RSBs. Although the study was successful, it was not without challenges. Several limitations were apparent in the process of conducting this study. When carrying out interviews and, especially given the sensitive nature of the topic in some societies, some participants, specifically secondary school students, were initially reluctant to participate, despite assurances of confidentiality and thorough explanations of the study’s purpose. Additionally, the research environment presented limitations, such as students feeling uncomfortable discussing SRH issues due to their status as students, even if they were engaging in related practices. This hesitancy to connect their experiences with what is taught could have affected the study’s outcomes.

## Conclusion

The disparities in the relevance of curriculum-based SRH education among students and teachers reveal a gap in utilizing and presenting comprehensive knowledge that will equip students with the right attitudes and skills needed in the current context, which demands awareness of healthy sexuality and the ability to properly approach RSBs. The available information from curriculum-based SRH education has forced secondary school students to improve upon the amount of information given by putting into practice what they are taught. Therefore, curriculum-based SRH education should include more comprehensive information to better equip secondary school students with appropriate skills to cope with RSBs.

## Data Availability

No datasets were generated or analysed during the current study.
